# Empirical assessment of competitive hybridization and noise in ultra high density canine tiling arrays

**DOI:** 10.1186/1471-2105-14-231

**Published:** 2013-07-22

**Authors:** Cali E Willet, Laura Bunbury-Cruickshank, Diane van Rooy, Georgina Child, Mohammad R Shariflou, Peter C Thomson, Claire M Wade

**Affiliations:** 1 Faculty of Veterinary Science, The University of Sydney, Sydney, NSW 2006, Australia; 2 University of Sydney Veterinary Teaching Hospital, The University of Sydney, Sydney, NSW 2006, Australia

**Keywords:** Array comparative genome hybridization, aCGH, Copy number variation, CNV, Tiling path offset, Oligonucleotide probe, Probe competition, *Canis lupus*

## Abstract

**Background:**

In addition to probe sequence characteristics, noise in hybridization array data is thought to be influenced by competitive hybridization between probes tiled at high densities. Empirical evaluation of competitive hybridization and an estimation of what other non-sequence related features might affect noisy data is currently lacking.

**Results:**

A high density array was designed to a 1.5 megabase region of the canine genome to explore the potential for probe competition to introduce noise. Multivariate assessment of the influence of probe, segment and design characteristics on hybridization intensity demonstrate that whilst increased density significantly depresses fluorescence intensities, this effect is largely consistent when an ultra high density offset is applied. Signal variation not attributable to sequence composition resulted from the reduction in competition when large inter-probe spacing was introduced due to long repetitive elements and when a lower density offset was applied. Tiling of probes immediately adjacent to various classes of repeat elements did not generate noise. Comparison of identical probe sets hybridized with DNA extracted from blood or saliva establishes salivary DNA as a source of noise.

**Conclusions:**

This analysis demonstrates the occurrence of competitive hybridization between oligonucleotide probes in high density tiling arrays. It supports that probe competition does not generate random noise when it is maintained across a region. To prevent the introduction of noise from this source, the degree of competition should be regulated by minimizing variation in density across the target region. This finding can make an important contribution to optimizing coverage whilst minimizing sources of noise in the design of high density tiling arrays.

## Background

Oligonucleotide-based microarrays, initially developed for the analysis of gene expression, have become popular cost-effective tools for other applications including detection of copy number variations (CNVs), DNA methylation, single nucleotide polymorphism (SNP) genotyping and sequence enrichment for high-throughput sequencing. Refined technology has enabled the precise application of millions of probes per array, affording researchers the freedom to explore entire genomes for novel features or to interrogate specific regions of interest with high coverage. For targeted analysis of putative CNV regions, probes are densely tiled adjacent to or overlapping one another according to predetermined spacing constraints [1,2]. This high density coverage enables smaller CNVs to be detected with enhanced statistical power [3] and with more precise localization of CNV breakpoints [4].

Although high density tiling offers a theoretical resolution of one base pair [5], in practice coverage is limited by two main factors. These are the presence of repetitive elements and characteristics of DNA sequence that prevent adequate probe sensitivity. Probes with non-unique sequence generate experimental noise through non-specific cross-hybridization, inhibiting data analysis and meaningful conclusions [2]. The sensitivity of a probe in successfully hybridizing and retaining target fragments is related to probe-target thermodynamics and propensity to form internal secondary structures [6]. Obtaining dense probe coverage is a challenge for probe design, as the goals of coverage, specificity and sensitivity are often incongruent [6,7].

In the avoidance of repetitive sequence in tiling array design, two main approaches exist. Low complexity and repetitive sequences can be filtered, generating a series of discontinuous unique segments to which probes are tiled to the desired density [1]. Alternatively, the entire region of interest can be tiled as a continuous string, retaining probes which pass some uniqueness threshold when scanned against the target genome database [2]. Whilst the latter approach may yield some coverage within regions traditionally annotated as repetitive, and avoid selection of non-unique probe sequences which escape the masking process due to unidentified species-specific repeats or presence in gene families, this technique relinquishes the ability to dictate probe coverage within the target region. If the former approach to avoiding repetitive sequence is adopted, the desired probe coverage can be enforced, yet a number of selected probe sequences may have short interspersed matches throughout the genome, potentially giving rise to experimental noise. In addition, an ‘edge effect’ is created, where the discontinuous segments are themselves tiled at a uniform density but are separated by potentially large distances, creating non-uniform density across the region as a whole.

In the optimization of high density array design, much research has been conducted investigating the influence of various probe characteristics on hybridization performance. Bertone *et al*. [1] focused on maximizing the number of unique bases within the target region that are covered by high specificity and sensitivity probes by manipulating probe length and melting temperature (Tm). Graf *et al*. [2] developed the uniqueness score to aid selection of probes that are most unlike any other substring of the reference genome. Sharp *et al*. [8] conducted a series of univariable analyses to determine the possible effects of probe uniqueness, presence of SNPs and homopolymers within the probe sequence, probe length and probe Tm on probe performance. Similarly, Flibotte *et al*. [9] determined that the number of off-target 15 base pair matches of probe sequences to the reference genome significantly influenced probe performance, as well as Tm, self-folding energy and presence of homopolymers. More recently Mulle *et al*. [10] conducted a multivariable assessment of a range of probe sequence characteristics and found that probe Tm, presence of SNPs and homocytosine motifs significantly influenced probe hybridization capacity.

Together these studies and others have established a set of design criteria for minimizing noise in tiling arrays by focusing on probe sequence. To the best of our knowledge, other aspects of array design specifically relating to the characteristics of the underlying target region have not yet been investigated. These include the length of and distance between discontinuous tiled segments, the positioning of and distance between probes within these segments, and the length and nature of repeat elements that separate tiled regions. We hypothesize that these variables may influence competitive hybridization between probes, another potential contributor to noise. This is a feature uniquely relevant to high density tiling arrays, where multiple probes are likely to share complimentarity for the same DNA fragments due to their close proximity in genomic space. Although DNA concentrations are applied to arrays in excess to minimize such effects, the vast number of identical probe sequences immobilized within each array feature suggests that given sufficient density, probe competition may ensue [11]. Stochastic factors such as the fluidic motion of DNA in solution and feature layout would then be expected to affect the proportion of available fragments that are bound by the competing features. This random noise would manifest as low repeatability of hybridization intensities from replicate probes, and may contribute to the observation that higher density arrays tend to yield noisier data. Higher density designs have also been observed to generate overall depression of signals [5], suggesting probe competition is a relevant factor. We aimed to empirically establish what factors may contribute to noise in high density tiling arrays and from this provide some guidelines for future custom high density designs.

We theorized that the presence of probe competition would be indicated by low repeatability of duplicate probes, significantly depressed hybridization intensity for probes tiled at higher density, to longer segments and towards the center of tiled segments, and significantly elevated hybridization intensity for probes tiled at the edge of unique segments of DNA that are adjacent to long un-tiled regions. We aimed to test these hypotheses through multivariable analysis of CGH array data from an unfiltered ultra high density probe set tiled to a repeat-masked region of the canine reference genome.

## Methods

### DNA samples

DNA samples were collected from a total of eight dogs (*Canis lupus familiaris*) belonging to two breeds. Peripheral blood samples were obtained from six dogs using EDTA blood collection tubes (BD Vacutainer, BD Franklin Lakes, NJ) and extracted using EZ1^®^ DNA Blood Kits (Qiagen, Valencia, CA). The remaining two samples were collected using Oragene ANIMAL OA-400 saliva collection kits (DNA Genotek, Ontario Canada) and extracted following standard kit-issued protocol.

Animal ethics approval for this project was granted by the University of Sydney Animal Ethics Committee (approval number N00/9-2009/3/5109).

### Probe design

Probes of 60 nucleotides in length (60mers) were designed using Perl v.5.10.1 to a 1.5 megabase (Mb) region of the canine reference genome sequence (canFam2.0, [12]). Interspersed repeats and low complexity sequences were filtered with RepeatMasker [13]. The region, spanning bases 13,400,000 – 14,900,000 of chromosome 38, was divided into three segments. Probes interrogating the region 14,000,000-14,200,000 were tiled with a 6 bp offset and the surrounding two regions with a 26 bp offset. In the event that a 60mer extended into a repeat-masked region, this probe was discarded. No further filtering was applied. Within the 6 bp offset tiled region, the outermost 5’ probe of segments at least 114 bp in length (the minimum length to achieve the maximum per-base probe coverage for 60mers at this offset) were replicated an additional four times.

### Microarray platform

Probes were synthesized in situ to one Agilent Custom 8 × 60 k oligonucleotide microarray (Agilent Technologies, Santa Clara, CA). Random feature layout was selected to minimize spatial autocorrelation. The standard set of Agilent control probes was included. Array hybridization and feature extraction was performed by the Ramaciotti Centre at the University of New South Wales in accordance with the manufacturer’s recommended protocol. A single-channel hybridization was carried out, where each sample was hybridized in isolation to a single array upon the slide, generating raw intensity values for each probe for each sample.

### Statistical analysis

Each probe-array combination was treated as a separate data point, yielding 410,280 data points in the unfiltered data set. Unlike two-channel arrays, where the ratio of test to reference hybridization is observed, the raw data in this experiment was the median signal for each probe. This single-channel data enabled the behavior of replicate probes to be compared between all eight genomic DNA samples, without interference with the raw results from competitive hybridization with a reference DNA sample. Demonstration of repeatability of probes in independent hybridizations was intended to support exploration of the factors, both known and of interest in this paper, that contribute to the wide range of hybridization intensities observed in CGH experiments.

### Probe competition

To assess the repeatability of hybridization intensity for replicated probes, variance was partitioned using ANOVA and repeatability (*r*) calculated according to the formula:

(1) $$r=\frac{S_{A}^{2}}{S^{2}+S_{A}^{2}}$$

where $S_{A}^{2}$ is the between group variance and *S*^2^ is the within group variance. Repeatability of the 94 custom probe sequences replicated five times each and of the 199 Agilent control probes with replication ranging from two to 252 was calculated for each sample separately.

The impact of probe and design variables on hybridization intensity was assessed by fitting a linear mixed model with splines using a restricted maximum likelihood (REML) procedure with GenStat 15^th^ Edition [14]. Spline terms were included for predictor variables which displayed non-linear relationships with fluorescence intensity (Additional file 1:A-D). A linear model was fitted to all remaining terms where no demonstrable non-linear relationships were observed (Additional file 1:E-N).Variables previously identified in the published literature as significant to probe performance were included to account for the influence of these known sources prior to evaluating potential competitive effects. These included:

• Probe GC: percentage of G and C nucleotides within the probe sequence. GC was used rather than Tm as all probes were of identical length, hybridized at the same salt and thermal conditions, and a correlation of 0.966 between GC and Tm has been previously reported [10].

• Off-target matches: number of off-target perfect matches in the reference genome of all 25mers contained within the probe sequence.

• Self-folding capacity: predicted free energy of the probe sequence in kcal/mol.

• Homopolymers: length of the longest homopolymer greater than 3 bp of each base.

• Background: background fluorescence associated with each array feature.

Design variables relating to probe competition are as follows:

• Offset: distance between the starting points of adjacent probes within tiled segments.

• Segment length: length in bp of the underlying tiled segment of unique reference DNA.

• Probe position: position of the probe within the tiled segment, with position numbers increasing towards the centre of the segment.

• Distance: distance across a repeat-masked region between two adjacent tiled segments.

The number of off-target matches was determined by scanning all probe sequences against a canFam2.0 25mer count database generated with Jellyfish v. 1.1.5 [15]. The free energy of each probe sequence was estimated with UNAFold [16] at the array hybridization temperature of 65°C. Probes tiled within two indicated CNV regions were removed from the analysis. Intensity values were log_*e*_-transformed to satisfy the assumption of normality and some explanatory variables were also log_*e*_-transformed to reduce influence of extreme values. Outliers were removed based on Agilent’s positive and negative control probes. Probes with background intensity greater than 50 were removed from the analysis to prevent undue influence of large outliers on the results. The explanatory variables of probe, dog and DNA source were included as random effects to adjust for variation in signal intensity from these anticipated sources. Separate models were required to evaluate the effects of probe position within segment (determined for every probe) and distance between tiled segments (calculated only for the first and last probes within a segment), since all probes with a valid value for distance (*n* = 2,907) were restricted to the value of 1 for position. The full linear mixed models which included the terms Position (Equation 2) and Distance (Equation 3) are shown below:

(2) $$\begin{array}{ll} {\text{log}}_{e}\text{Intensity}= & \beta_{0}+\beta_{1}\text{ProbeGC}+\beta_{2}\text{OffTargets}\\ & +\beta_{3}\text{FreeEnergy}+\beta_{4}\text{PolyC}+\beta_{5}\text{PolyG}\\ & +\beta_{6}\text{PolyA}+\beta_{7}\text{PolyT}+\beta_{8}l{\mathrm{og}}_{e}\text{Background}\\ & +\beta_{9}l{\mathrm{og}}_{e}\text{SegmentLength}+\beta_{10}\text{Position}\\ & +\text{Offset}+s\left(\text{PolyC}\right)+s(\text{PolyG})+s(\text{PolyA})\\ & +s(\text{PolyT})+\text{Probe}+\text{Dog}+\text{DNAsource}\\ & +\epsilon \end{array}$$

(3) $$\begin{array}{ll} {\text{log}}_{e}\text{Intensity}= & \beta_{0}+\beta_{1}\text{ProbeGC}+\beta_{2}\text{OffTargets}\\ & +\beta_{3}\text{FreeEnergy}+\beta_{4}\text{PolyC}+\beta_{5}\text{PolyG}\\ & +\beta_{6}\text{PolyA}+\beta_{7}\text{PolyT}+\beta_{8}l{\mathrm{og}}_{e}\text{Background}\\ & +\beta_{9}l{\mathrm{og}}_{e}\text{SegmentLength}+\beta_{10}{\text{log}}_{e}\text{Dis}\text{tan}\mathrm{ce}\\ & +\text{Offset}+s\left(\text{PolyC}\right)+s(\text{PolyG})+s(\text{PolyA})\\ & +s(\text{PolyT})+\text{Probe}+\text{Dog}+\text{DNAsource}\\ & +\epsilon \end{array}$$

where ProbeGC, OffTargets, FreeEnergy, PolyC, PolyG, PolyA, PolyT, Background and SegmentLength and Position are fixed covariate effects, Offset is a fixed factor effect, Probe, Dog and DNAsource are random effects and ϵ is the residual error. Spline terms, added into the random model, were indicated by the *s*(·) terms in the equations above. Variables were treated as independent based on the absence of correlation amongst the predictor variables extreme enough to cause collinearity (Additional file 2). A variable was considered to have a significant impact on hybridization intensity if the associated *P*-value was less than 0.05.

To gain an indication of signal variance between each offset unbiased by the magnitude of the mean, the coefficient of variation was computed for the filtered hybridization intensities in the copy number neutral regions analyzed above. To remove influence of DNA source, only the six blood sample arrays were used for the computation of coefficient of variation.

### Comparison of blood and saliva arrays

To determine if hybridization intensities differed significantly between DNA samples sourced from blood or saliva, unpaired t-tests were performed using GenStat. Probe intensities within the copy number neutral region including probes tiled at both offsets were analyzed. The coefficient of variation was computed for each DNA source group.

### Influence of repeat elements on adjacent probe hybridization

The unbound tails of DNA fragments hybridized to probes theoretically have the capacity to secondarily hybridize to other DNA fragments. Probes adjacent to repeat-masked regions may display increased fluorescence intensity due to this phenomenon, with higher-copy repeat types likely affected to a greater extent. To determine if the nature of a nearby repetitive element influences hybridization intensity, all repeat elements within an 80 kb region shown not to be involved in a CNV and spanning both offsets were examined. The effects of the class and length of the repeat along with the probe and design variables identified as significant in earlier analyses were analyzed using REML to assess their influence on probe intensity. The number of off-target matches was not considered as all probes within this probe set had zero off-target matches. Where multiple classes of repeat were present within the one repeat-masked region, the class of the longest repeat and the combined length of all repeats was recorded. In the 6 bp offset region the closest four probes to each repetitive element were included, and the closest two probes in the 26 bp offset region. By forming a distinct subset of probes from within the larger filtered dataset, a separate model was required to estimate the effects of repeat class and length. The linear mixed model for the repeat element analysis is shown below:

(4) $$\begin{array}{ll} {\text{log}}_{e}\text{Intensity}= & \beta_{0}+\beta_{1}\text{ProbeGC}+\beta_{2}\text{FreeEnergy}\\ & +\beta_{3}\text{PolyC}+\beta_{4}\text{PolyG}+\beta_{5}\text{PolyA}\\ & +\beta_{6}\text{PolyT}+\beta_{7}l{\mathrm{og}}_{e}\text{Background}\\ & +\beta_{8}l{\mathrm{og}}_{e}\text{RepeatLength}+\mathrm{RepeatClass}\\ & +\text{Offset}+s\left(\text{PolyC}\right)+s\left(\text{PolyG}\right)+s\left(\text{PolyA}\right)\\ & +s\left(\text{PolyT}\right)+\text{Probe}+\text{Dog}+\text{DNAsource}\\ & +\epsilon \end{array}$$

where ProbeGC, OffTargets, FreeEnergy, PolyC, PolyG, PolyA, PolyT, Background and RepeatLength are fixed covariate effects, RepeatClass and Offset are fixed factor effects, Probe, Dog and DNAsource are random effects and ϵ is the residual error, with the *s*(·) terms indicating splines as above.

### Effects of probe sequence deletions on hybridization

Hybridization intensities of the deletion stringency control probes (DCP), a standard component of Agilent’s control probe set, were analyzed to determine the extent to which deleted bases from the center of a probe sequence affect hybridization. As the exact control probe sequences were unavailable, probes were grouped according to number of deleted central nucleotides (0–8) and graphically summarized as boxplots using R v. 2.9.1 [17]. To compare the intensities of probes with eight deleted central nucleotides to the negative controls, an unpaired *t*-test was conducted using the blood sample array data.

## Results

A total of 30,669 probes were tiled at an offset of 26 bp and 20,616 at an offset of 6 bp. Designed probes interrogated 65.23% of the nucleotides within the target region. 0.09% of probes failed, returning intensities less than the negative controls and 1.95% of probes showed intensities greater than the positive controls. 7.84% of these showed complete feature saturation, where the maximum raw intensity of 65,530 was recorded for that probe. Variables and intensities are summarized in Table 1.

**Table 1:** Summary of probe and design variables and hybridization intensities for eight arrays

**Variable**	**Minimum value**	**Maximum value**	**Median value**
Raw intensity	44	65530	395
Background intensity	26	653.5	36
Positive control intensity	70	15103	9346.75
Negative control intensity	39	135	57.5
Probe GC (%)	11.67	86.67	36.67
Free energy (kcal/mol)	-9.48	3.8	0.48
Off-target matches	0	4496	0
PolyA length (bp)	0	19	3
PolyC length (bp)	0	17	0
PolyG length (bp)	0	19	0
PolyT length (bp)	0	19	3
Segment length (bp)	61	5760	1156
Distance (bp)	6	7791	190.5
Offset (bp)	6	26	na
Probe position	1	389	na

### Probe competition

Repeatability of designed replicates ranged from 0.989 to 0.998 for blood sample arrays and 0.986 and 0.998 for salivary DNA arrays. Perfect repeatability to at least six decimal places was demonstrated for the Agilent control replicates.

**Figure 1:**
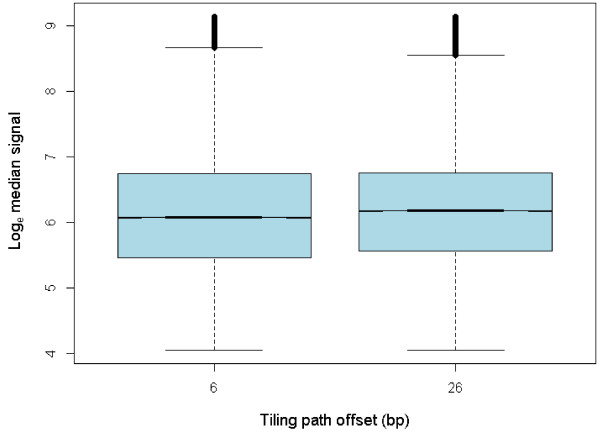
**Log**_**e **_**median signal by tiling path offset (bp). **Two medians are significantly different at the 5% level if their notches do not overlap. Blood sample array data shown.

Following filtering of probes with intensity above or below the positive and negative control medians respectively, with background greater than 50 and exclusion of those residing in identified CNV regions, 47,711 probes remained for each array, giving a total of 381,688 data points in the probe position model and 22,392 in the distance model. Probes tiled at an offset of 6 bp fluoresced significantly lower than those tiled at a 26 bp offset (*P* < 0.001, Table 2, Table 3, Figure 1). The coefficients of variation for 6 bp and 26 bp offsets were 1.395% and 1.295%, respectively.

**Table 2:** REML output for the probe position dataset

**Fixed term**	***F *statistic**	***P*-value**	**Effect**	**Standard error**
Probe GC	17334.54	<0.001	0.069	0.0005
log_e_ Off-targets	152.34	<0.001	0.460	0.037
Free energy	1626.64	<0.001	0.153	0.004
PolyA	33.81	<0.001	0.034	0.006
PolyC	7.30	0.008	0.081	0.030
PolyG	37.21	<0.001	0.132	0.022
PolyT	101.30	<0.001	0.050	0.005
Offset	650.03	<0.001		0.007^a^
6 bp			0	
26 bp			0.190	
log_e _Background	364.97	<0.001	0.142	0.007
log_e _Segment length	17.84	<0.001	-0.019	0.004
Probe position	4.04	0.044	0.0002	0.00009

^a ^Standard error of the difference between the effect of 6 bp or 26 bp offset.

This probe set included all probes, after filtering. The predicted effects of each variable and factor level on hybridization intensity (log_e _scale) are shown and the associated standard error of predicted effects.

**Table 3:** REML output for the distance dataset

**Fixed term**	***F *statistic**	***P*-value**	**Effect**	**Standard error**
Probe GC	976.27	<0.001	0.068	0.002
log_e _Off-targets	14.49	<0.001	0.421	0.111
Free energy	100.23	<0.001	0.165	0.016
PolyA	5.05	0.030	0.036	0.016
PolyC	9.20	0.005	0.167	0.056
PolyG	13.16	0.012	0.100	0.027
PolyT	13.94	<0.001	0.058	0.016
Offset	31.25	<0.001		0.037^a^
6 bp			0	
26 bp			0.210	
log_e _Background	15.28	<0.001	0.124	0.032
log_e _Segment length	2.11	0.146	-0.019	0.013
log_e _Distance	3.96	0.047	0.024	0.012

^a ^Standard error of the difference between the effect of 6 bp or 26 bp offset.

This probe set included the first and last tiled probe within each tiled segment, after filtering. The predicted effects of each variable and factor level on hybridization intensity (log_e _scale) are shown and the associated standard error of predicted effects.

**Figure 2:**
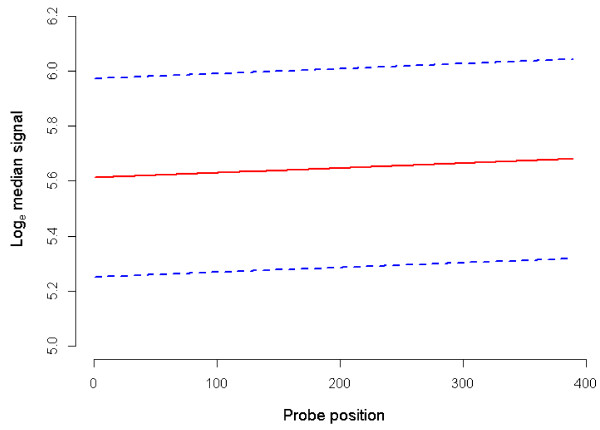
**Predicted mean log**_**e **_**median signal by probe position. **Mean log_e _median signal at each level of probe position when all REML covariates are held constant at the mean and averaged over all factor levels (solid line). Dashed lines indicate the mean log_e_ median signal +/- standard errors of the predictions.

**Figure 3:**
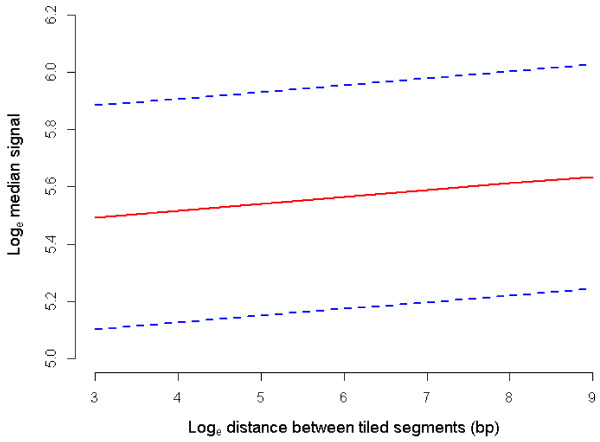
**Predicted mean log**_**e **_**median signal by log**_**e **_**distance between tiled segments (bp). **Mean log_e _median signal at each level of log_e _distance when all REML covariates are held constant at the mean and averaged over all factor levels (solid line). Dashed lines indicate the mean log_e _median signal +/- standard errors of the predictions.

**Figure 4:**
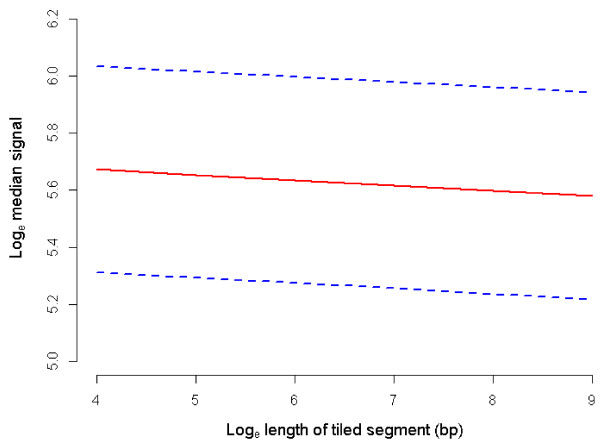
**Predicted mean log**_**e **_**median signal by log**_**e **_**length of tiled segment (bp). **Mean log_e _median signal at each level of log_e _length of tiled segment when all REML covariates are held constant at the mean and averaged over all factor levels (solid line). Dashed lines indicate the mean log_e _median signal +/- standard errors of the predictions.

Position of probe was found to have a significant linear effect on hybridization intensity (*P* = 0.044, Table 2, Figure 2), with each unit increase in probe position associated with an increase in fluorescence intensity of 0.0002 ± 0.00009 on the log_e_ scale. Increasing distance in bp between tiled segments displayed a significant positive linear effect on hybridization intensity for probes tiled at the edges of segments (*P* = 0.047, Table 3, Figure 3), with each additional bp separating tiled segments increasing signal from these probes by 0.024 ± 0.012 on the log_e_ scale. Length of tiled segment did not significantly influence hybridization capacity of probes tiled at the edges of segments (Table 3), but did have a significant negative linear effect on intensity when all probes within a segment were considered (*P* < 0.001, Table 2; Figure 4, REML estimated effect -0.019 ± 0.004 on the log_e_ scale).

All remaining terms in the Distance and Probe Position models were found to have a significant linear influence on fluorescence intensity (Tables 2,3). Increasing homopolymer lengths of the nucleotides A, C, G and T was also found to have significant non-linear effects on intensity in the Probe Position model (*P* < 0.001, χ^2^=89.78, d.f.=1; *P* < 0.001, χ^2^=164.17, d.f.=1; *P* < 0.001, χ^2^=283.59, d.f.=1; *P* < 0.001, χ^2^=166.8, d.f.=1, respectively) (Additional file 3:A-D) and the Distance model (*P* = 0.013, χ^2^=6.23, d.f.=1; *P* < 0.001, χ^2^=18.11, d.f.=1; *P* = 0.05, χ^2^=283.59, d.f.=1; *P* = 0.002, χ^2^=166.8, d.f.=1, respectively) (Additional file 3:E-H).

### DNA source

The mean hybridization intensity of the saliva sample arrays (390.46) was significantly lower than the mean of blood sample arrays (793.77) (*P* < 0.001; *t* = 146.68; d.f. = 292). Variances were estimated separately due to significant *F* tests of unequal variances (*P* < 0.001; *F* = 3.05; n.d.f. = 289; d.d.f. = 96). The coefficients of variation for probe intensities measured using blood and saliva samples were 1.308% and 1.496%, respectively.

### Influence of repeat elements on adjacent probe hybridization

Within the analyzed 80 kb region where no CNV had been identified, a total of 82 repeat-masked regions were observed and grouped according to repeat class based on UCSC Genome Browser (http://genome.ucsc.edu/) annotation: Long interspersed nuclear elements (LINEs), short interspersed nuclear elements (SINEs), long terminal repeats (LTR), simple repeats (Simple) and low-complexity sequence (LC). There were 474 probes passing filtering within this region, yielding 3,792 unique probe-array combinations in the data subset. Neither the class (dropped from the full fixed model) nor length of repetitive elements (*P* = 0.736, Table 4) were found to have an observable linear effect on the fluorescence intensity of adjacent probes in the region analyzed.

**Table 4:** REML output for the repeat element dataset

**Fixed term**	***F *statistic**	***P*-value**	**Effect**	**Standard error**
Probe GC	121.40	<0.001	0.051	0.005
Free energy	8.56	0.004	0.109	0.038
PolyA	0.75	0.389	0.022	0.025
PolyC	19.64	<0.001	0.235	0.053
PolyG	5.74	0.017	0.045	0.019
PolyT	2.89	0.121	-0.021	0.028
Offset	4.51	0.034		0.059^a^
6 bp			0	
26 bp			0.124	
log_e _Background	0.58	0.446	-0.022	0.028
log_e _Repeat length	0.11	0.736	0.013	0.038

^a ^Standard error of the difference between the effect of 6 bp or 26 bp offset.

### Effect of probe sequence deletions on hybridization

**Figure 5:**
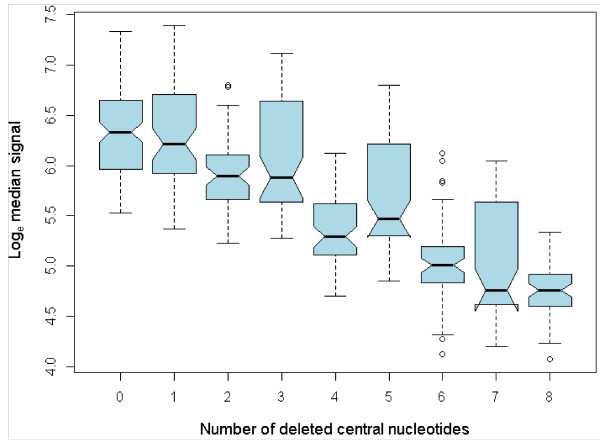
**Log**_**e **_**median signal by number of deleted central nucleotides. **20 sets of Agilent DCP probes were printed onto each array. Each set comprised 10 probes: two replicate parent probes (non-deleted, 0) and four replicated probes with one, three, five or seven deletions or two, four, six or eight deletions depending on the sequence set. Two medians are significantly different at the 5% level if their notches do not overlap Blood sample array data shown.

The mean intensity for the DCP probes tended to decrease with an increasing number of deleted central nucleotides (Figure 5). DCP probes with five deleted central nucleotides retained an average of 57% of the non-deleted signal, whilst probes with eight deletions retained an average of 24% of the parent signal and displayed significantly greater mean intensity (121.67) than the mean of negative controls (69.87 ) (*P* < 0.001; *t* = 11.356; d.f. = 60).

## Discussion

To the authors’ knowledge, this study presents the first empirical evaluation published in the peer reviewed literature of probe competition as a source of noise in high density tiling arrays. A multivariable analysis is used to demonstrate that whilst increased density depresses signals overall, this effect is not random but is influenced by the degree of competition between probes within a region. High repeatability of replicate probes supports the absence of random noise from high density tiling. Comparison of identical arrays hybridized with DNA extracted from blood or saliva provides what is to our knowledge the first indication that salivary DNA can contribute to noise in aCGH. This study also establishes that probe characteristics known to be influential in human tiling arrays are also important in canine aCGH experiments. The results presented here will be an important contribution to the design of future high density tiling arrays for achieving maximum coverage and minimum noise.

An advantage of single channel aCGH as utilized in this experiment is the ability to compare the data from multiple arrays. This enabled data pooling for multivariable analysis, and the lack of probe filtering provided a dataset with a range of characteristics for statistical assessment. Within the 98% of probes remaining after filtering, variation unexplained by CNV was observed. This is consistent with the observation that increased density in CGH arrays decreases the signal to noise ratio [4,5,8,18]. If the null hypothesis that this noise was in part due to competition between probes held true, low concordance between replicated probes printed at random locations across the arrays would be expected. This was not the case, with probe repeatability across a range of replicated probe sequences greater than 0.98 for all samples. Confidence in the repeatability of signals from duplicate probes underlies the basis of exploring what factors, if not random noise, lead to the wide variation in signal intensity from oligonucleotide probes. Such an understanding will enable these factors to be taken into account when calling CNVs from CGH data.

Mixed effect modeling was carried out to investigate the possibility of probe competition after accounting for the influence of known contributors to probe performance. As anticipated, probe GC, free energy, homopolymers of each of the four nucleotides, and background fluorescence all had a significant positive linear influence on signal intensity (Table 2, Table 3). A significant non-linear effect was also observed for homopolymers of A,C,G and T nucleotides. Whilst the biological explanation for this is difficult to comment on from the available data, it does assert the importance of avoiding long homopolymers in oligonucleotide probe sequences where possible.

In determining a significant influence of the number of 25 bp off-target matches to hybridization, our results demonstrate that as little as 42% of the length of the probe sequence is capable of binding and retaining DNA fragments. This phenomenon was corroborated by the ability of 60mers with up to eight bases deleted from the center of the oligonucleotide to display significantly higher signal than the negative controls. This highlights the benefits of assessing probe uniqueness according to probe *k-*mers rather than relying on repeat masking or whole-probe alignments. As laboratories trend towards whole genome sequencing, mining existing CNV data rather than generating new arrays will likely rise in popularity. Our finding that over 4,000 off-target matches may be present within a single theoretically unique probe advocates the application of *k*-mer filtering of historical probe sets to aid avoidance of false positive signals.

A further observation that can be made from the deletion analysis is that the presence of short insertion deletion (indel) polymorphisms are unlikely to cause substantially reduced hybridization capacity on this platform. This suggests that probe design need not avoid known indels. The interesting pattern depicted in Figure 5, where an odd number of deleted nucleotides tended to reduce hybridization capacity to a greater extent than even-numbered deletions, is difficult to comment on given the unavailability of Agilent’s control probe sequences. This phenomenon warrants further investigation with known control sequences to increase our understanding of hybridization between imperfectly matched DNA and oligonucleotide sequences.

In our assessment of probe competition, increasing the tiling path offset was found to have a significant linear effect on fluorescence intensity. Probes tiled at 6 bp demonstrated lower intensity compared to 26 bp (*P* < 0.001, Table 2, Table 3, Figure 1). Although this result is limited to two offsets differing by just 20 bp, a 200 bp segment tiled at 6 bp offset will be interrogated with 23 probes compared to five probes at 26 bp offset, demonstrating a meaningful difference between competitive effects at the offsets tested. The similar coefficients of variation support that this observed increase in competitive hybridization at 6 bp tiling offset did not generate random noise. Further, all duplicated probes displayed almost perfect repeatability. Taken together, these results suggest that competition between probes from high density tiling has a general dampening influence on hybridization intensity without introducing stochastic variation.

For probes designed to the border between tiled and repeat-masked sequence, the density of probes and thus degree of competition is reduced. This was shown by the significant elevation in signal for probes at the edge of longer untiled regions (*P* = 0.047, Table 3, Figure 3). For these probes, the length of the segment and thus increase in number of neighbor probes was not influential given the lack of competition to one side of the genomic neighborhood. In contrast, when all probes within a segment were considered, longer tiled segments tended to bear probes with significantly lower hybridization intensity (*P* < 0.001, Table 2, Figure 4). This increased competition affected probes within segments to a somewhat consistent degree, with probe position having only a small effect on intensity (*P* = 0.044, Table 2, Figure 2). Our results support the existence of competitive hybridization that does not itself contribute to random noise, but the level of which is subject to variation due to the discontinuous nature of unique sequence within genomes.

This edge effect, where probes designed to regions adjacent to long untiled regions display increased signals from reduced competition, may be minimized by applying the method described by Graf *et al*. [2]. Designing probes within repeat-masked regions through the use of *k*-mer uniqueness thresholds will not only dampen the edge effect, but may also moderate the fluctuation in competition across the region in general. This approach is unlikely to introduce noise through secondary hybridization of repetitive sequence, given our finding that neither the class nor the length of repeat elements in close proximity to probes affected hybridization intensity. To corroborate this notable finding, our analysis of 474 probe sequences across 80 kb of genomic DNA should be repeated with a larger number of probes across more diverse genomic regions.

The inclusion of DNA samples extracted from two different sources enabled opportunistic assessment of the influence of DNA source on aCGH data. Intensities obtained from saliva arrays were significantly lower than those from blood and demonstrated a higher coefficient of variation. This increase in noise may result from inadequate target DNA concentration due to microbial contamination. Up to 90% of DNA extracted from Oragene saliva samples has been found microbial in origin [19]. Inadequate input DNA limits the number of fragments available for hybridization, leading to variable capture amongst probes competing for the same sequences. Given that our conclusions are drawn from two salivary DNA arrays, validation of this finding is recommended. On the basis of our results alone, if the use of salivary DNA in aCGH experiments is unavoidable it is recommended that a species-specific DNA quantification assay be implemented to ensure the recommended concentration is met. In the post-hoc analysis of publicly available aCGH data, comparisons between single-channel arrays should be matched for DNA source where possible and salivary two-channel arrays potentially analyzed in a less stringent fashion to avoid false negatives from weak signals.

While funding constraints prevented our confirmation of these results in other genomic regions, we anticipate these results are applicable to the canine genome in general. The chosen region was representative of the reference with respect to GC content, presence of exonic, intronic and intergenic sequence, and comprised various repeat classes common to the canine genome. Further experiments tiling probes to other genomic regions and hybridizing a greater number of samples is still warranted to corroborate these findings. Of particular interest is testing a greater number of tiling path offsets and replicating these levels of offset at multiple locations across the genome.

## Conclusions

In what we believe is the first analysis of this type, our results empirically establish the significant dampening effect of competitive hybridization between probes tiled at high densities. Our near-perfect probe repeatability supports that this effect is not stochastic. The finding that variations in the degree of competition through large untiled regions or long densely tiled stretched does influence hybridization intensity demonstrates a need to moderate competitive effects. Since analysis of CNV from CGH data involves ratios rather than raw intensities, depression in intensity from probe competition does not affect CNV detection when the competitive effect is consistent across the array. This can be achieved without increasing noise by tiling probes with minimal off-target matches to traditionally repeat-masked regions. Our study also indicates that inadequate DNA concentration can introduce noise by increasing competition amongst probes for limited complimentary DNA fragments. The findings of this study provide important array design insights for CNV detection where the ability to densely tile probes without increasing noise is favorable for both precision and statistical power.

### Availability of supporting data

The raw data files supporting the results of this article (http://www.ebi.ac.uk/arrayexpress/experiments/E-MEXP-3860/) and the associated array design (http://www.ebi.ac.uk/arrayexpress/arrays/A-MEXP-2294/) are available in the ArrayExpress repository.

## Competing interests

The authors declare that they have no competing interests.

## Authors’ contributions

CEW designed the tiling array, performed the variable calculation, carried out the statistical analyses and prepared the manuscript. LBC, DvR and GC collected the samples. MS performed the DNA extraction and helped to edit the manuscript. PT assisted with statistical analyses and helped to edit the manuscript. CMW conceived of the study, participated in its design and coordination and helped to edit the manuscript. All authors read and approved the final manuscript.

## Supplementary Material

Additional file 1 Exploratory data analysis: Relationships between response and predictor variables. Click here for file

Additional file 2 Exploratory data analysis: Relationships amongst predictor variables. Click here for file

Additional file 3 Non-linear relationships between homopolymer length of the four nucleotide bases and the response variable. Click here for file
