# Supplementary material for: Empirical assessment of competitive hybridization and noise in ultra high density canine tiling arrays
Source: BMC Bioinformatics. 2013 Jul 22;14:231. doi: 10.1186/1471-2105-14-231 (PMC3733988; doi:10.1186/1471-2105-14-231)
Supplement: Additional file 1 — Exploratory data analysis: Relationships between response and predictor variables. [file 1471-2105-14-231-S1.docx]

**A Additional File 1**

**Exploratory data analysis: Relationships between response and predictor variables**

**A**


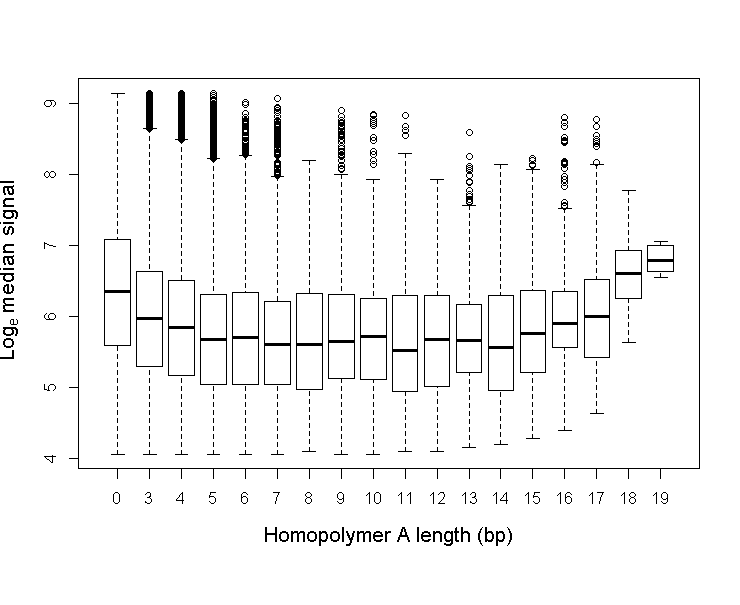


**B**


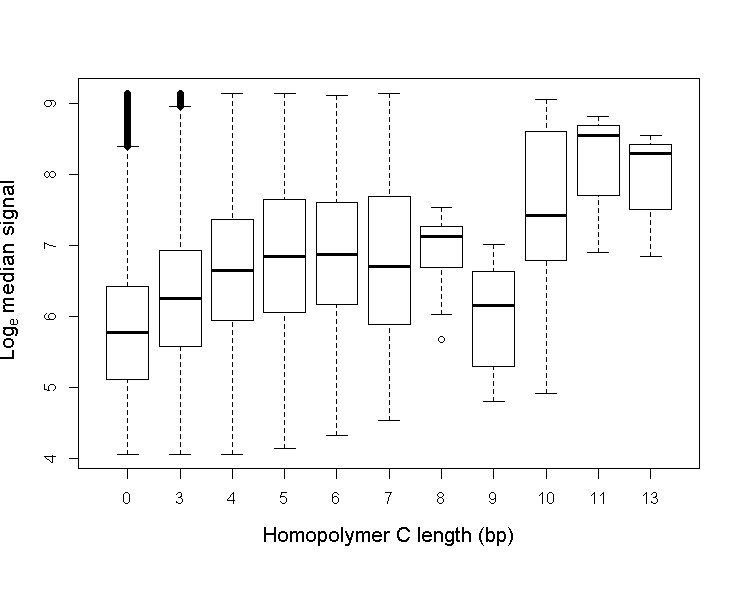


**C**

**
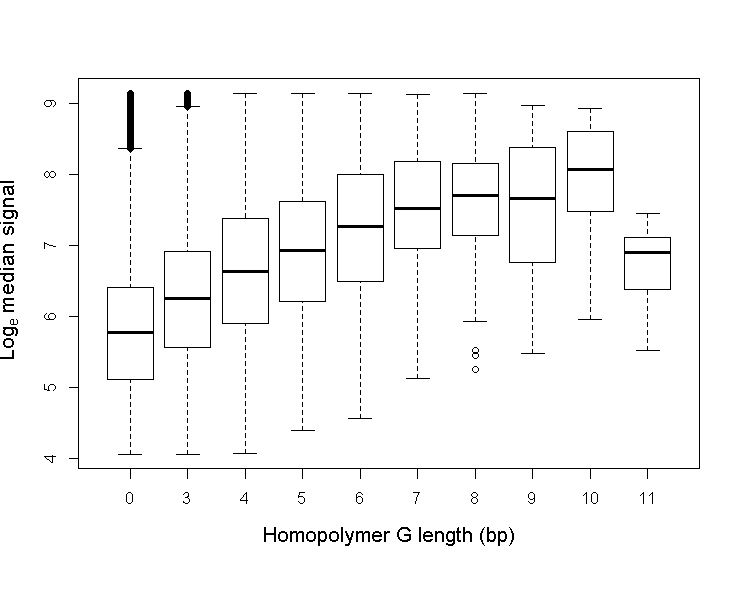
**

**D**


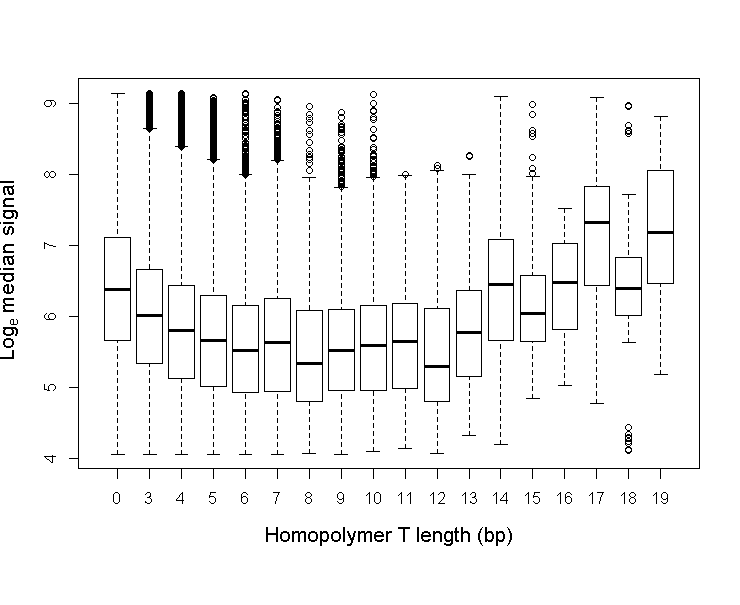


**E**


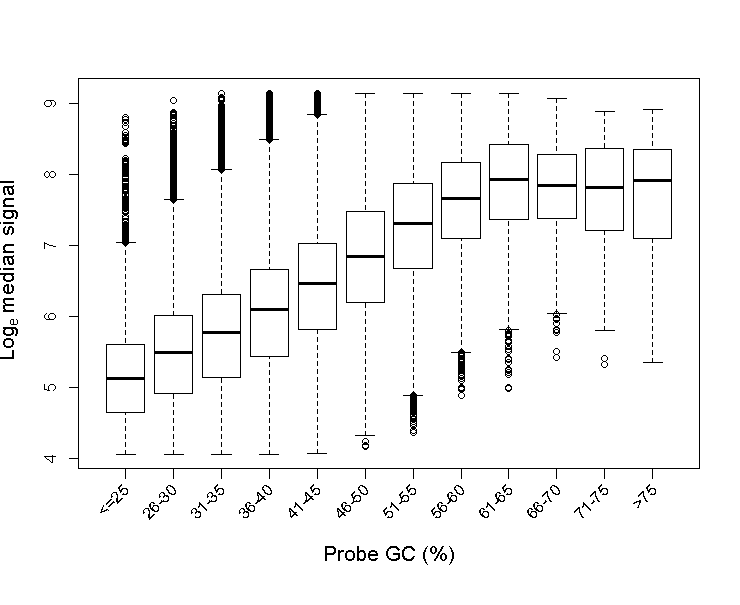


**F**


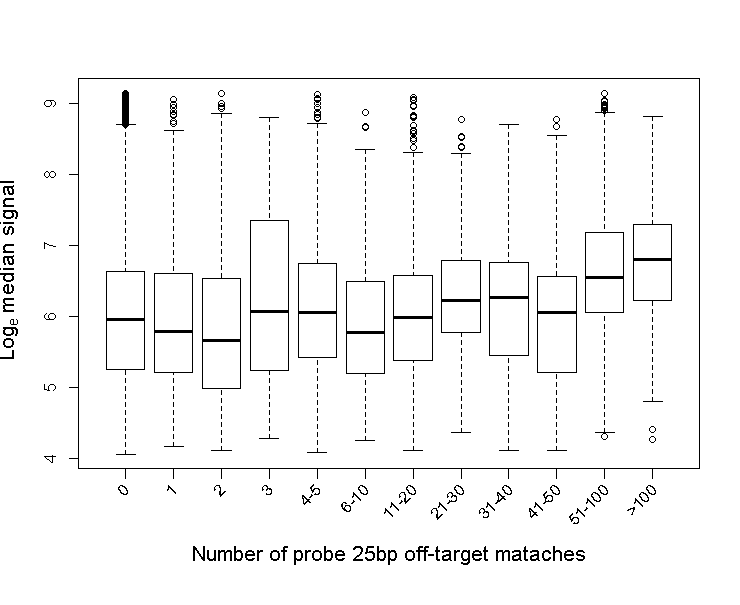


**G**


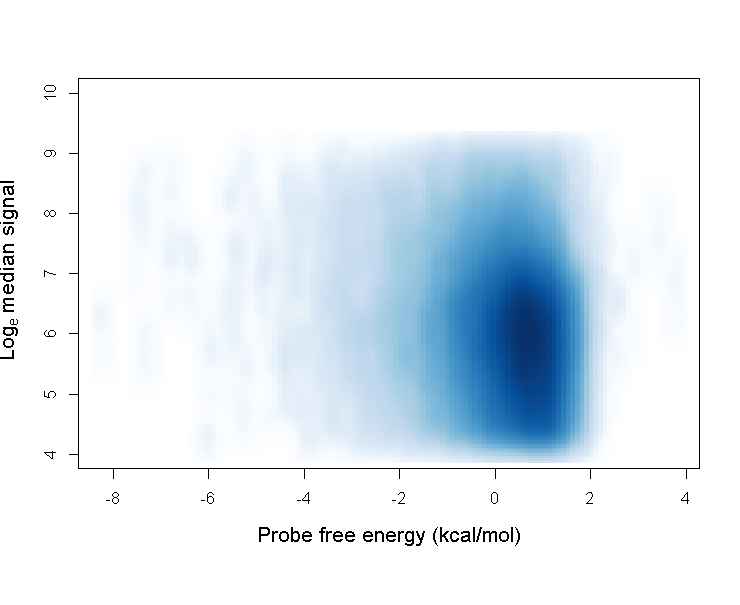


**H**


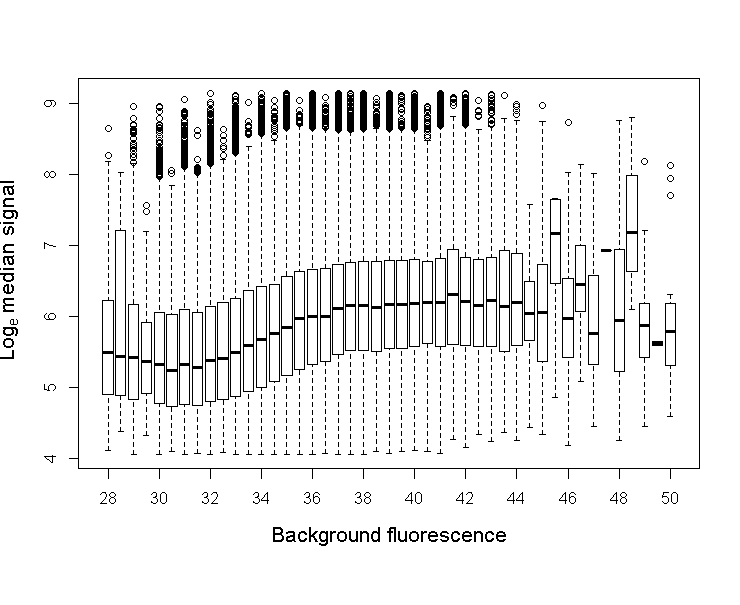


**I**


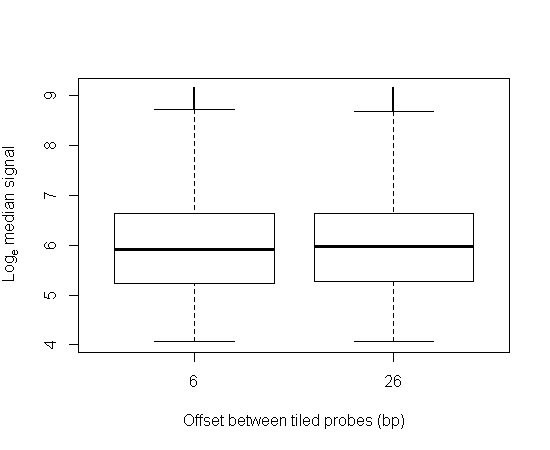


**J**


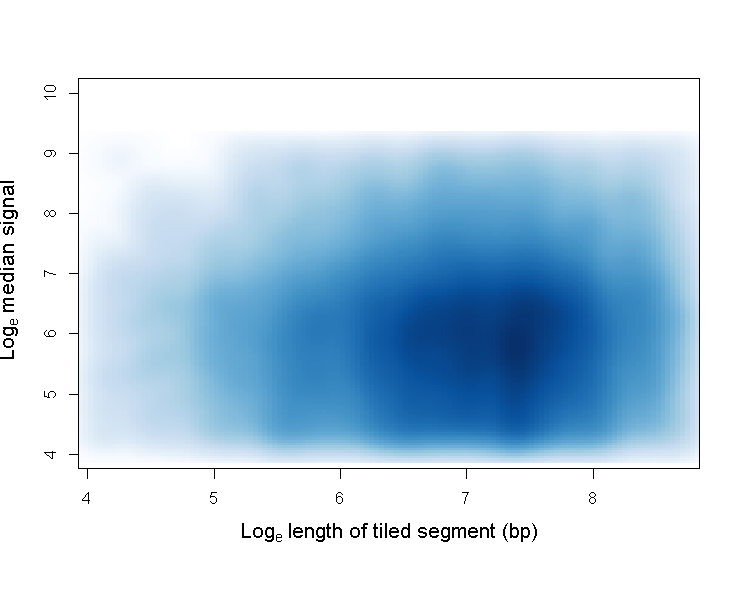


**K**


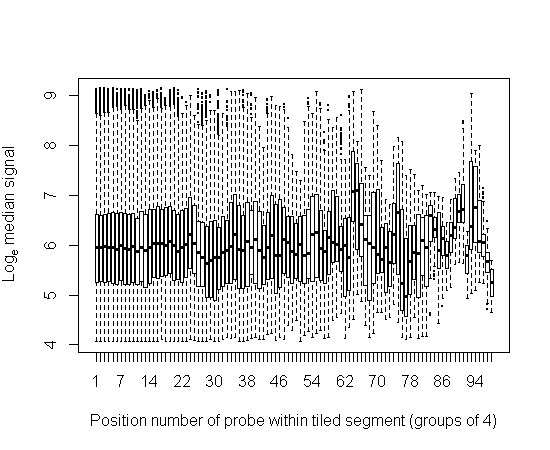


**L**


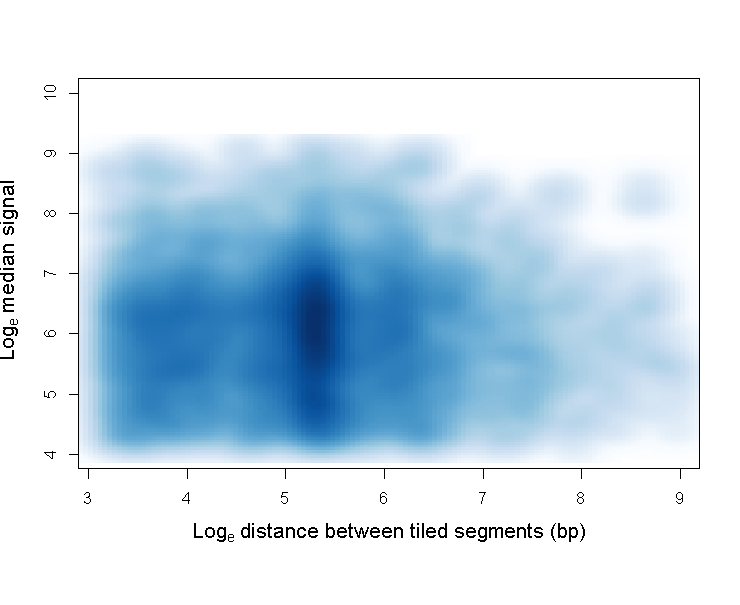


**M**


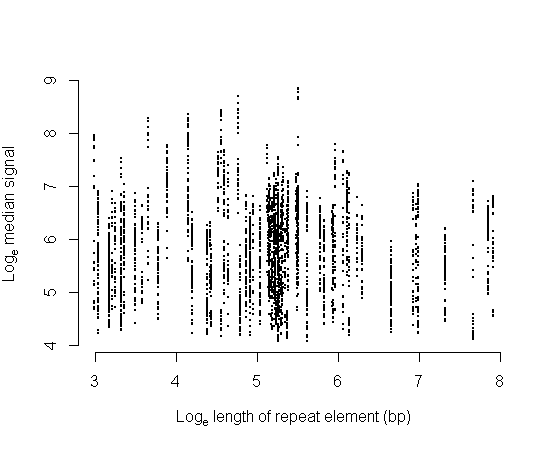


**N**

**
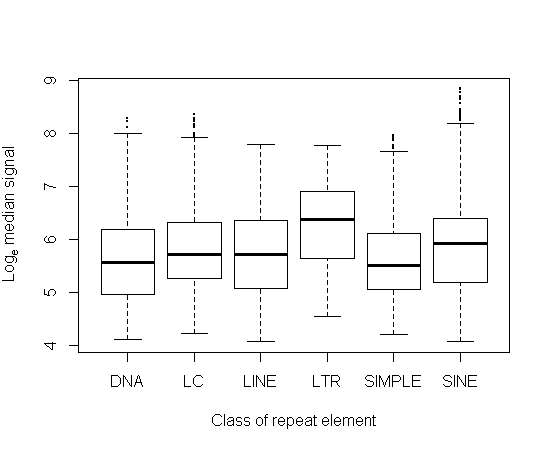
**

Figure A.1. Exploratory data analysis of the relationship between the response variable log_e_ median signal and each predictor variable.

A Log_e_ intensity by the length in base pairs of the longest adenine homopolymer within the probe sequence.

B Log_e_ intensity by the length in base pairs of the longest cytosine homopolymer within the probe sequence.

C Log_e_ intensity by the length in base pairs of the longest guanine homopolymer within the probe sequence.

D Log_e_ intensity by the length in base pairs of the longest thymine homopolymer within the probe sequence.

E Log_e_ intensity by probe GC range (%).

F Log_e_ intensity by the number of 25bp probe substring off-target matches to the canFam2.0 [1] reference genome sequence. The number of substring matches was determined using Jellyfish v. 1.1.5 [2].

G Log_e_ intensity by probe free energy expressed in kilocalories per mole. Free energy was calculated for each probe at the array hybridization temperature of 65 degrees Celsius using UNAFold [3].

H Log_e_ intensity by background fluorescence intensity. Probes with background intensity greater than 50 were removed from the dataset to prevent undue influence of these large outliers on the results.

I Log_e_ intensity by the number of base pairs offset between the genomic starting positions of adjacent tiled probes.

J Log_e_ intensity by log_e_ length in base pairs of the tiled segment of genomic reference DNA.

K Log_e_ intensity by position of probe within tiled segment. Probes tiled at the 5’ and 3’ edge of the segment are allocated position 1, with position numbers increasing towards the centre of the tiled segment. For clarity, positions have been grouped, with positions 1 through 4 allocated group 1, positions 5 through 8 allocated group 2 and so on.

L Log_e_ intensity by log_e_ distance in base pairs between adjacent tiled segments of genomic reference DNA.

M Log_e_ intensity by log_e_ length in base pairs of the repeat element or cluster of repeat elements separating adjacent tiled segments.

N Log_e_ intensity by the class of repeat element separating tiled segments of unique genomic reference DNA.

**References**

[1] Lindblad-Toh K, Wade CM, Mikkelsen TS, Karlsson EK, Jaffe DB, Kamal M, Clamp M, Chang JL, Kulbokas EJ, Zody MC *et al*: **Genome sequence, comparative analysis and haplotype structure of the domestic dog**. *Nature* 2005, **438**(7069):803-819.

[2] Marcais G, Kingsford C: **A fast, lock-free approach for efficient parallel counting of occurrences of k-mers**. *Bioinformatics* 2011, **27**(6):764-770.

[3] Markham NR, Zuker M: **UNAFold : Software for nucleic acid folding and hybridization**. *Methods Mol Biol* 2008, **453**:3-31.
