# Supplementary material for: Empirical assessment of competitive hybridization and noise in ultra high density canine tiling arrays
Source: BMC Bioinformatics. 2013 Jul 22;14:231. doi: 10.1186/1471-2105-14-231 (PMC3733988; doi:10.1186/1471-2105-14-231)
Supplement: Additional file 2 — Exploratory data analysis: Relationships amongst predictor variables. [file 1471-2105-14-231-S2.docx]

**A Additional File 2**

**Exploratory data analysis: Relationships amongst predictor variables**

**A**


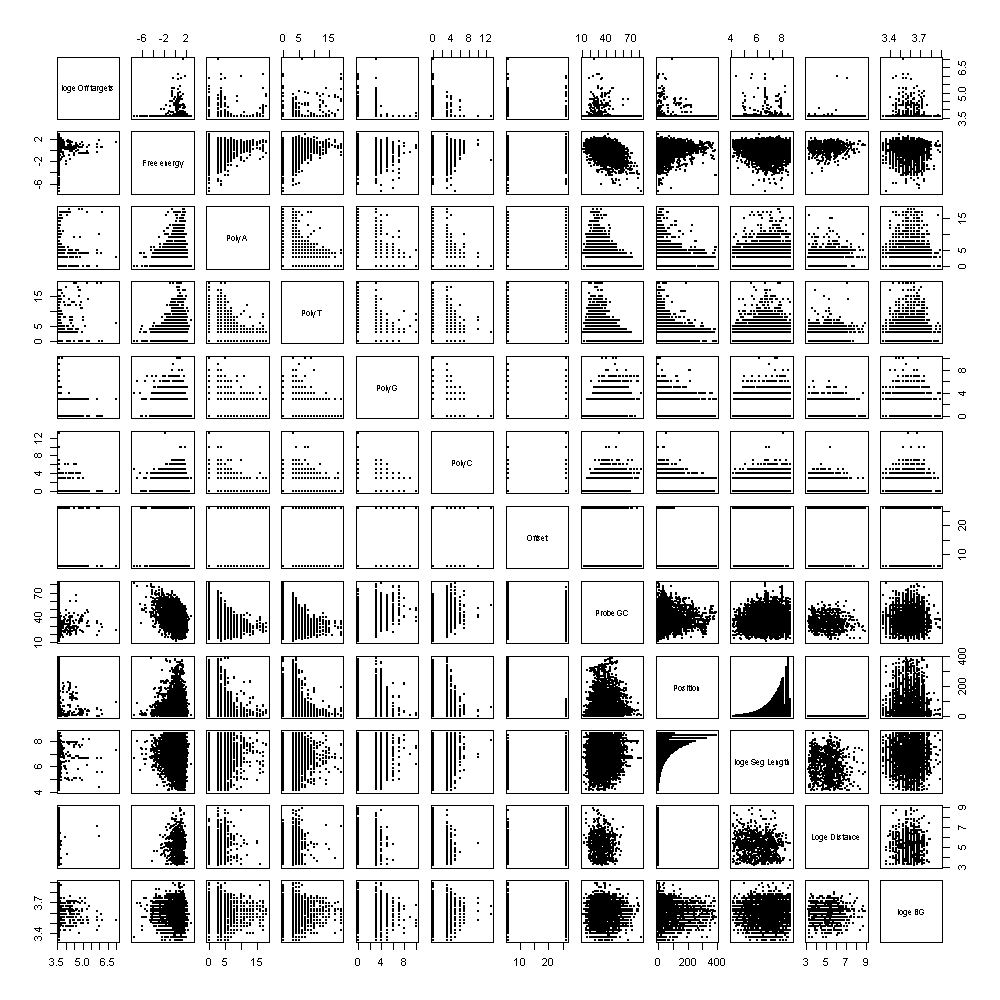


**B**

|  | log_e_ Off-targets | Free energy | Poly A | Poly T | Poly G | Poly C | Offset | Probe GC | Probe position | log_e_ Segment length | log_e_ Distance |
| --- | --- | --- | --- | --- | --- | --- | --- | --- | --- | --- | --- |
| log_e_ Off-targets | 1 | 0.030 | 0.058 | 0.064 | -0.025 | -0.014 | -0.051 | -0.083 | 0.034 | 0.010 | 0.009 |
| Free energy | 0.030 | 1 | 0.119 | 0.168 | -0.214 | -0.100 | 0.061 | -0.399 | -0.064 | -0.033 | 0.039 |
| Poly A | 0.058 | 0.119 | 1 | -0.019 | -0.097 | -0.124 | 0.075 | -0.392 | -0.037 | 0.006 | 0.036 |
| Poly T | 0.064 | 0.168 | -0.019 | 1 | -0.138 | -0.069 | -0.007 | -0.390 | -0.022 | -0.021 | -0.023 |
| Poly G | -0.025 | -0.214 | -0.097 | -0.138 | 1 | 0.022 | -0.076 | 0.380 | 0.045 | -0.002 | 0.000 |
| Poly C | -0.014 | -0.100 | -0.124 | -0.069 | 0.022 | 1 | -0.026 | 0.336 | 0.036 | 0.030 | -0.022 |
| Offset | -0.051 | 0.061 | 0.075 | -0.007 | -0.076 | -0.026 | 1 | -0.121 | -0.504 | -0.006 | 0.093 |
| Probe GC | -0.083 | -0.399 | -0.392 | -0.390 | 0.380 | 0.336 | -0.121 | 1 | 0.121 | 0.061 | -0.043 |
| Probe position | 0.034 | -0.064 | -0.037 | -0.022 | 0.045 | 0.036 | -0.504 | 0.121 | 1 | 0.588 | NA |
| log_e_ Segment length | 0.010 | -0.033 | 0.006 | -0.021 | -0.002 | 0.030 | -0.006 | 0.061 | 0.588 | 1 | -0.104 |
| log_e_ Distance | 0.009 | 0.039 | 0.036 | -0.023 | 0.000 | -0.022 | 0.093 | -0.043 | NA | -0.104 | 1 |

Figure A.2. Exploratory data analysis of the relationships amongst the predictor variables.

A Scatter plot of relationships amongst predictor variables. A random sub-sample of 20,000 data points was obtained from the full dataset using the ‘sample’ function in R [1] and plotted using the ‘pairs’ function.

B Spearman’s correlation coefficients amongst numeric predictor variables. Correlations were computed using the ‘cor’ function in R [1] between all complete pairs of observations on those variables. No correlation could be computed between distance and probe position because all probes with non-missing values for distance were probe position 1.

**References**

[1] R Core Development Team: R: **A Language and Environment for Statistical Computing**. R Foundation for Statistical Computing, Vienna, Austria; 2009.
