# Supplementary material for: Empirical assessment of competitive hybridization and noise in ultra high density canine tiling arrays
Source: BMC Bioinformatics. 2013 Jul 22;14:231. doi: 10.1186/1471-2105-14-231 (PMC3733988; doi:10.1186/1471-2105-14-231)
Supplement: Additional file 3 — Non-linear relationships between homopolymer length of the four nucleotide bases and the response variable. [file 1471-2105-14-231-S3.docx]

**A Additional File 3**

**Non-linear relationships between homopolymer length of the four nucleotide bases and the response variable**

**A**

**
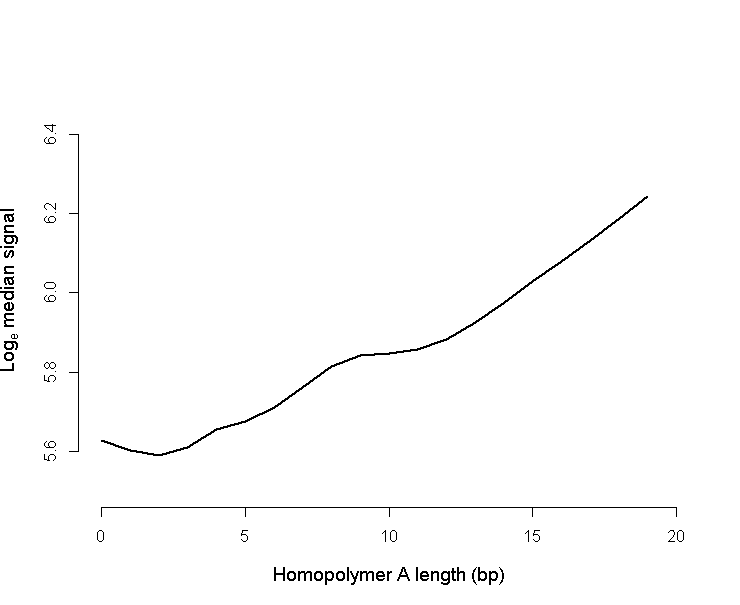
**

**B**

**
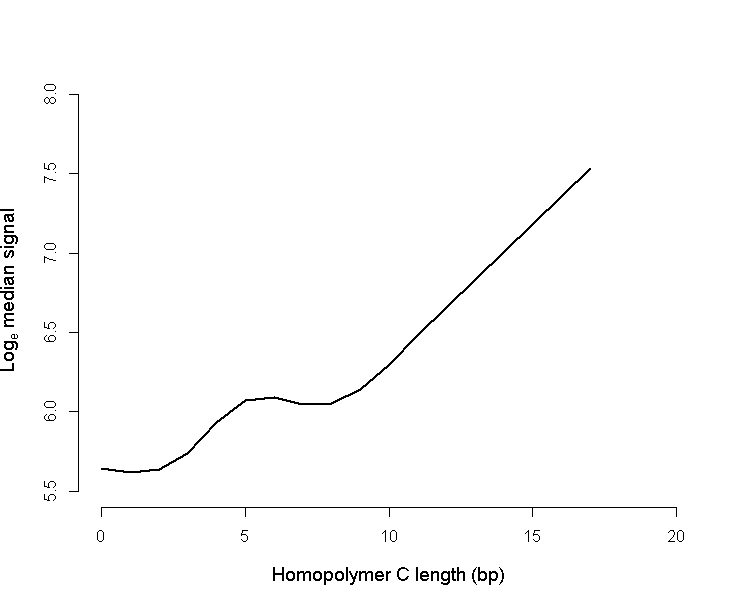
**

**C**

**
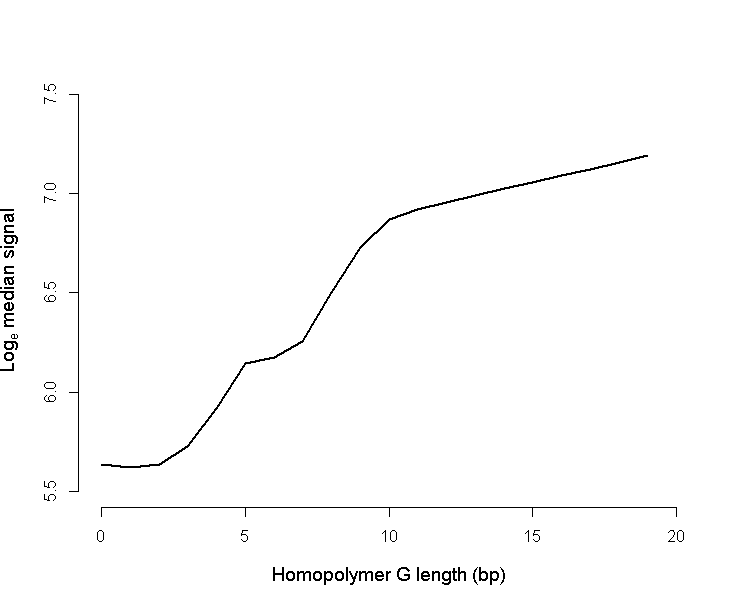
**

**D**

**
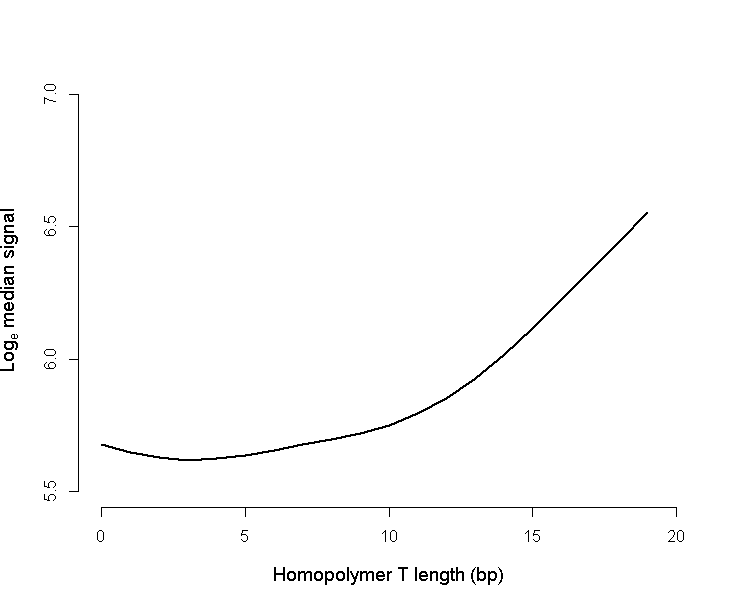
**

**E**

**
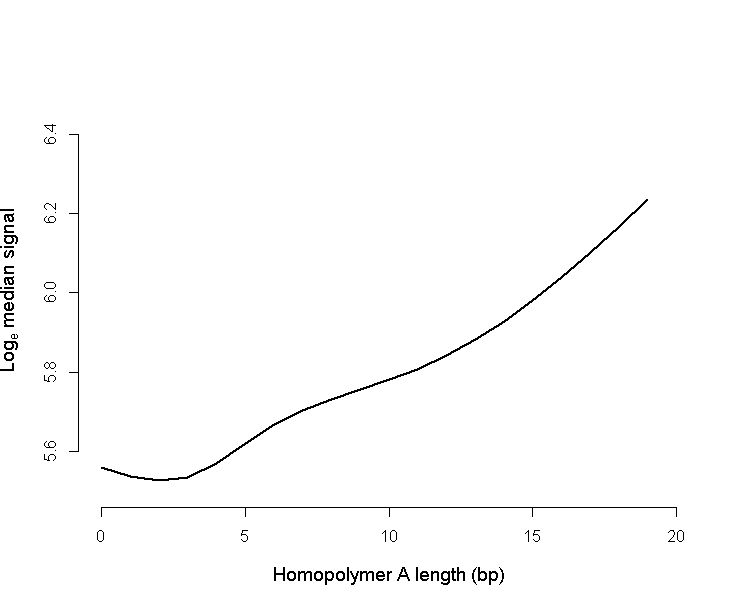
**

**F**

**
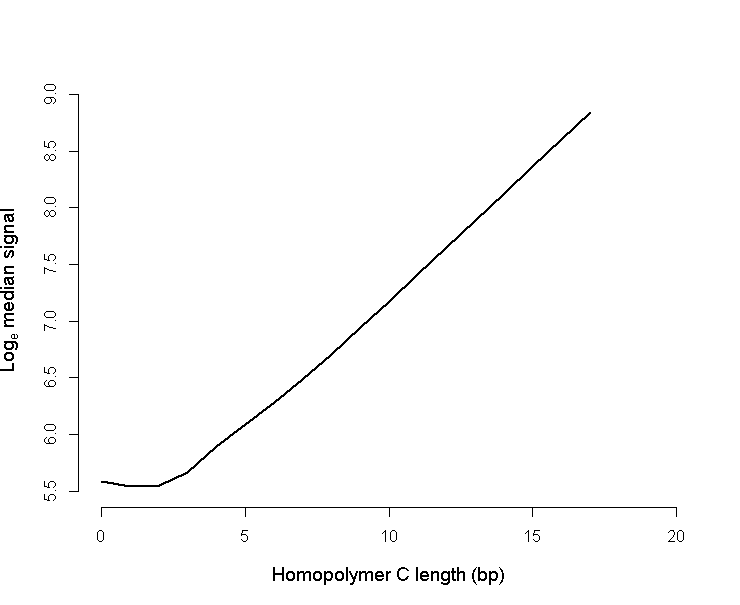
**

**G**

**
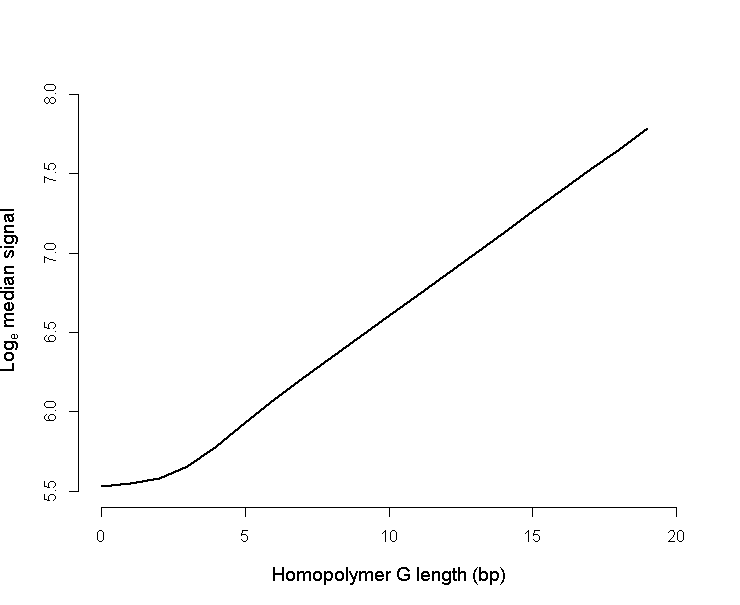
**

**H** **
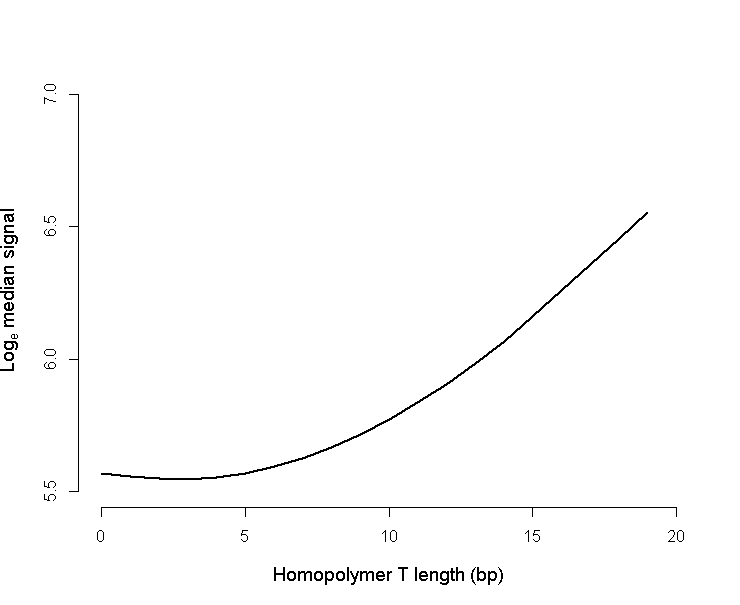
**

Figure A.3. Mean log_e_ median signal at each level of homopolymer length when all REML covariates are held constant at the mean and averaged over all factor levels.

A Mean log_e_ median signal by the length in base pairs of the longest adenine homopolymer within the probe sequence for all probes within the probe position dataset.

B Mean log_e_ median signal by the length in base pairs of the longest cytosine homopolymer within the probe sequence for all probes within the probe position dataset.

C Mean log_e_ median signal by the length in base pairs of the longest guanine homopolymer within the probe sequence for all probes within the probe position dataset.

D Mean log_e_ median signal by the length in base pairs of the longest thymine homopolymer within the probe sequence for all probes within the probe position dataset.

E Mean log_e_ median signal by the length in base pairs of the longest adenine homopolymer within the probe sequence for all probes within the distance dataset.

F Mean log_e_ median signal by the length in base pairs of the longest cytosine homopolymer within the probe sequence for all probes within the distance dataset.

G Mean log_e_ median signal by the length in base pairs of the longest guanine homopolymer within the probe sequence for all probes within the distance dataset.

H Mean log_e_ median signal by the length in base pairs of the longest thymine homopolymer within the probe sequence for all probes within the distance dataset.
